# The perspective of medical staff on improving the integrated communication process – A pilot study

**DOI:** 10.25122/jml-2021-0186

**Published:** 2021

**Authors:** Mirela Morcov, Iuliana-Raluca Gheorghe, Maria Veronica Morcov, Victor Lorin Purcarea

**Affiliations:** 1. Department of Quality Management and Healthcare Services, Dr. Nicolae Robanescu National Neurorehabilitation Center for Children, Bucharest, Romania; 2. Department of Healthcare Marketing and Medical Technology, Carol Davila University of Medicine and Pharmacy, Bucharest, Romania; 3. Department of Therapeutic Education, Dr. Nicolae Robanescu National Neurorehabilitation Center for Children, Bucharest, Romania; 4. Department of Recovery, Physical Medicine and Balneology, Carol Davila University of Medicine and Pharmacy, Bucharest, Romania

**Keywords:** communication, health services, medical team, quality assurance

## Abstract

The quality of working relationships among medical team members is influenced by communication, which has a significant impact on patient safety. Our study took place at the Dr. Nicolae Robanescu National Neurorehabilitation Center for Children, Bucharest, between October and December 2019 and included 44 participants that were grouped into three categories: physicians, physical therapists, and nurses (32 women and 12 men), aged between 23 and 53, all of whom were employed in the same unit. A total of 5 questions were used to select data on communication difficulties. The chi-square test used for statistical analysis revealed no significant differences between the professional categories participating in the research (p>0.05). We suggest increasing the group size in a future study to increase the statistical significance.

## Introduction

The challenge for most organizations in a global market characterized by high dynamism and fierce competition is to identify the most effective ways to communicate with consumers so they understand the benefits of purchasing and consuming services [1]. The increasing demands that medical service recipients express as a result of the advancement of medical culture and the connection to associated technologies in maintaining health place an increasing strain on medical organizations and, inevitably, their communication capacity.

Communication is the process by which a sender transfers information, intentionally or unintentionally, to at least one receiver, which can be reciprocal [2]. When we talk about health unit communication, we mean both internal communication (the link between medical staff and non-medical staff – support that leads to the proper conduct of activities) and external communication (with authorities, competition, service providers, community, non-governmental organizations – NGOs, but most importantly with the patient, which requires intense communication) [3].

The patient is the “main actor” in the health system because he is a pioneer in the mechanism of performing the medical act, collaborating with medical service providers to acquire high-quality health care [4]. Physicians are trained using different approaches from those used by nurses, which might lead to challenges in interprofessional communication (depending on each person’s personality) [5]. Moreover, communication between medical team members influences the quality of working relationships and has a significant impact on patient safety [6].

Effective communication at the organizational level necessitates open expression and listening on both sides [7]; it is the key to a mature and strong relationship in understanding the discussion partner and requires coherence, legitimacy, credibility, attractiveness, and admissibility [8]. However, in Romania, it has been observed that communication becomes difficult in an interdisciplinary context, with many limitations [9]. This study aims to compare three professions’ communication processes within an interdisciplinary team: physicians, physical therapists, and nurses. The objectives of the study are:

• To investigate the levels of communication and collaboration between employees in horizontal and vertical positions at the Dr. Nicolae Robanescu National Clinical Center for Neuropsychomotor Recovery (questions no. 5, 6, 7);
• To determine the level of professional communication training of employees at the Dr. Nicolae Robanescu National Clinical Center for Neuropsychomotor Recovery (question no. 11);
• To evaluate the level of understanding and collaboration among people in a higher level of authority (question no. 34).

## Material and Methods

### Participants

The study included 44 participants (32 women and 12 men), aged between 23 and 53, and took place in the same unit between October – December 2019. Physicians, physical therapists, and nurses were the specialists who took part in the study. The questions were explained to the subjects enrolled in the study, and informed consent was obtained for participation.

### Data collection tool

The questionnaire method (opinion questionnaire) was used to conduct the study, which was based on the questionnaire technique, which consists of a collection of logical and cohesive questions. The questionnaire contains 36 complex questions, of which only those related to the communication issue (5 questions) were chosen, maintaining the number assigned in the initial questionnaire.

The questionnaire’s content, which was administered using the “pencil-paper” method, was explained to the subjects. The chosen questions concerned employee communication and collaboration between the Center’s structures (the hierarchical superior – manager) and communication training; these are closed questions with 3–6 answer options. The study’s five questions are listed below.

5. How do you appreciate the communication and collaboration between the structures within Dr. Nicolae Robanescu National Clinical Center for Neuropsychomotor Recovery?

6. Is there a communication and collaboration relationship between you and your superior?

7. Do you consider that there is an effective communication and collaboration relationship between you as an employee and the manager of the organization?

11. Have you taken a medical practice communication course?

34. Do you consider that your supervisor and the management team (the manager and the financial accounting director) listen to you and consider your suggestions for improvement?

Four answer alternatives are available for question no. 5, and the respondent must choose one of the following:

a) Very satisfactory; b) Satisfactory; c) Less satisfactory; d) Unsatisfactory.

Question no. 6 and 7 have the following response options:

a) Yes; b) Sometimes; c) No.

Question no 11 has three possible answers:

a) More; b) One; c) None.

Question no. 34 has six answers (any of them may be chosen):

a) They consider my suggestions; b) They appreciate my opinions; c) They agree with my ideas; d) They listen to me; e) Sometimes; f) No.

### Statistical analysis

Data collection and processing were performed using the Statistical Package for the Social Sciences (SPSS) version 27. Frequencies and percentages were used to describe qualitative and nominal data. To highlight the difference between the groups, the X2 (Chi-square) test was used. The statistical significance level was set at 0.05. The hi square test was used for data processing.

## Results

### Demographic data

In total, 118 (62%) of the 189 (100%) employees of the Center are medical, paraclinical, and paramedical personnel (Table 1). The study included 44 specialists, representing 23.28% of total employment. The study did not include new employees or those on leave, and some refused to participate. The employees polled ranged in age from 23 to 53 years old, with a mean age of 41 years old (40 years old – physicians; 39 years old – physical therapists and 44 years old – nurses). Females made up 32 of the 44 respondents (72.73%) (Table 2). Furthermore, 32 of the 44 participants (72.73%) have higher education (Table 3).

**Table 1.** Distribution by professional category.

**Profesional category**	**Frequency**	**Percentage**
**Physician**	19	43.18
**Paramedical**	16	36.36
**Paraclinic**	9	20.46
**Total**	44	100.0

**Table 2.** Distribution by sex.

**Sex**	**Frequency**	**Percentage**
**Female**	32	72.73
**Male**	12	27.27
**Total**	**44**	**100.0**

**Table 3.** Distribution according to the level of education.

**Education Level**	**Frequency**	**Percentage**
**Medium**	12	27.27
**Superior**	32	72.73
**Total**	**44**	**100.0**

### Questionnaire results

**Figure 1.**
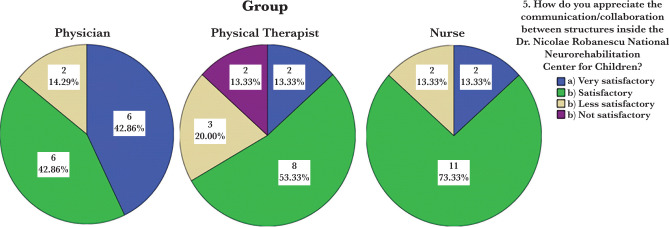
Percentage distribution of the staff related to communication and collaboration.

Only two employees chose the answer “(communication is) unsatisfactory”, while the majority of respondents (25 out of 44,56.8%) chose the option “(communication is) satisfactory” (Table 4). The statistical relevance of this question (calculated according to the algorithm described by Rampichini [10]) is 0.0825, and the weight assigned to the question is 15.76%. Because the relevance is small, we can conclude that the groups do not differ significantly from one another when only the answers to this question are considered (Table 5). It can also be seen that the proportions of the answers “(communication is) very satisfactory” in the “Physician” group are 42.8%, apparently higher than the analogous proportions in the “Physical therapist” and “Nurse” groups, respectively (both of 13.3%) (Figure 1).

**Table 4.** Frequency and percentage of the assessment of communication and collaboration between structures.

**5. How do you appreciate the communication and collaboration between the structures within the Dr. Nicolae Robanescu National Clinical Center for Neuropsychomotor Recovery?**	**Frequency**	**Percentage**
**a) Very satisfactory**	10	22.7
**b) Satisfactory**	25	56.8
**c) Less satisfactory**	7	15.9
**d) Unsatisfactory**	2	4.5
**Total**	**44**	**100.0**

**Table 5.** Assessment of communication and collaboration between structures by categories of employees.

**Question**		**5.How do you appreciate the communication and collaboration between the structures within the Dr. Nicolae Robanescu National Clinical Center for Neuropsychomotor Recovery?**				**Total**
		**a) Very satisfactory**	**b) Satisfactory**	**c) Less satisfactory**	**d) Unsatisfactory**	
**Group**	**Physician**	6	6	2	0	**14**
	**Physical Therapist**	2	8	3	2	**15**
	**Nurse**	2	11	2	0	**15**
	**Total**	10	25	7	2	**44**

However, given the small number of respondents in the groups, these differences are not statistically significant (p=0.093 given by the chi-square test, above the significance threshold of 0.05). Answers “No (there is a relationship...)” and “No (I consider that there is a relationship ...)” are relatively rare, representing only 2.3% and 4.5% of the total, respectively. The vast majority of respondents – about two-thirds for each question – answered “Yes (there is a relationship...)” and “Yes (I consider that there is a relationship...)” which would correspond to the quality criteria (Tables 6 and 7). The answers of personnel categories are shown in Tables 8 and 9. As presented in Table 9, negative answers are found only in the “Physical Therapist” group.

**Table 6.** Frequency and percentage of communication and collaboration relationship between employee and supervisor.

6. Is there a communication and collaboration relationship between you and your supervisor?	**Frequency**	**Percentage**
**a) Yes**	30	68.2
**b) Sometimes**	13	29.5
**c) No**	1	2.3

**Table 7.** Frequency and percentage of effective communication and collaboration between employee and manager.

**7. Do you consider that there is an effective communication and collaboration relationship between you as an employee and the manager of the organization?**	**Frequency**	**Percentage**
**a) Yes**	29	65.9
**b) Sometimes**	13	29.5
**c) No**	2	4.5
**Total**	**44**	**100.0**

**Table 8.** Communication and collaboration relationship between employee and supervisor by personnel categories.

Group	**6. Is there a communication and collaboration relationship between you and your superior?**			
	**a) Yes**	**b) Sometimes**	**c) No**	**Total**
**Physician**	11	3	0	14
**Physical Therapist**	9	5	1	**15**
**Nurse**	10	5	0	**15**
**Total**	30	13	1	**44**

**Table 9.** Effective communication and collaboration relationship between employee and manager by personnel categories.

**Group**	**7. Do you consider that there is an effective communication and collaboration relationship between you as an employee of the organization and the manager?**			
	**a) Yes**	**b) Sometimes**	**c) No**	**Total**
**Physician**	10	4	0	**14**
**Physical Therapist**	9	4	2	**15**
**Nurse**	10	5	0	**15**
**Total**	29	13	2	**44**

**Figure 2.**
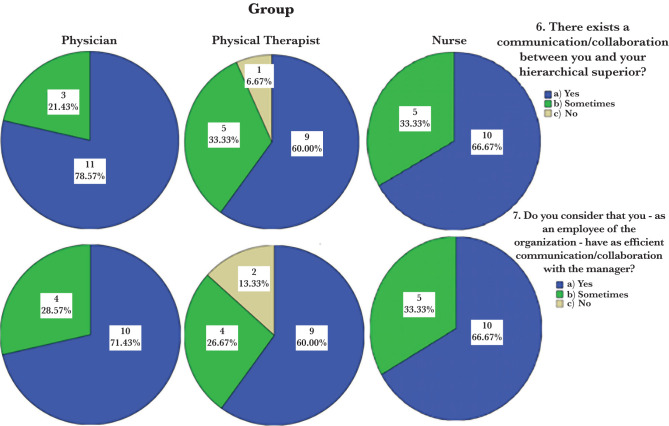
Employee percentage distribution in terms of communication and consultation relationships with the supervisor, as well as effective communication and collaboration with the manager.

Question no. 6 has a statistical relevance of 0.0284 (weighted at 5.43%), while question no. 7 has a statistical relevance of 0.023 (weight 4.40%) (Figure 2). According to the responses to these questions, the groups do not differ significantly from one another. When applied to question no. 6 (comparing the distributions of answers on the three groups), the Chi-square test showed a p-value of 0.556, indicating that the three groups’ answers are not statistically significantly different (but that they do not give similar answers).

**Figure 3.**
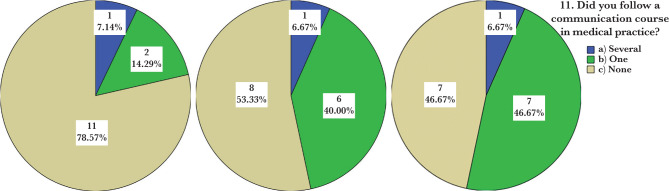
Percentage distribution of the number of communication courses in medical practice by professional categories.

In the case of question no. 7, the test produces a p-value of 0.807, which is close to 1, therefore suggesting that the three groups would provide similar answers. Table 10 summarizes the responses given to question no. 11. Only three employees chose the answer “More”, while the majority of respondents (26 out of 44, 59.1%) chose the answer “None”. The responses to the personal categories are presented in Table 11. The group responses “More” are evenly distributed. The medical staff provided the most “None” answers. Figure 3 shows that the proportions of the “None” answers in the “Physician” group are apparently higher compared to the “Physical Therapist” and “Nurse” groups.

**Table 10.** Number of participations in communication courses in medical practice by professional categories.

Group	**11. Have you taken any communication courses in medical practice?**			
	**a) Several**	**b) One**	**d) None**	**Total**
**Physician**	1	2	11	**14**
**Physical Therapist**	1	6	8	**15**
**Nurse**	1	7	7	**15**
**Total**	3	15	26	**44**

**Table 11.** Frequency and percentage of courses in medical practice.

11. Did you take a communication course in medical practice?	**Frequency**	**Percentage**
**a) More**	3	6.8
**b) One**	15	34.1
**c) None**	26	59.1
**Total**	**44**	**100.0**

For question no. 11, the calculated statistical relevance is 0.0605 (weight assigned – 11.56%), slightly higher than the previous questions but too small to indicate essential differences between groups (Figure 3). The Chi-square test applied to question 11 (comparing the distributions of the answers on the three groups) showed a p-value of 0.186, higher than the threshold of statistical significance (0.05). Therefore, we cannot say that the three groups’ answers are statistically significantly different.

**Figure 4.**
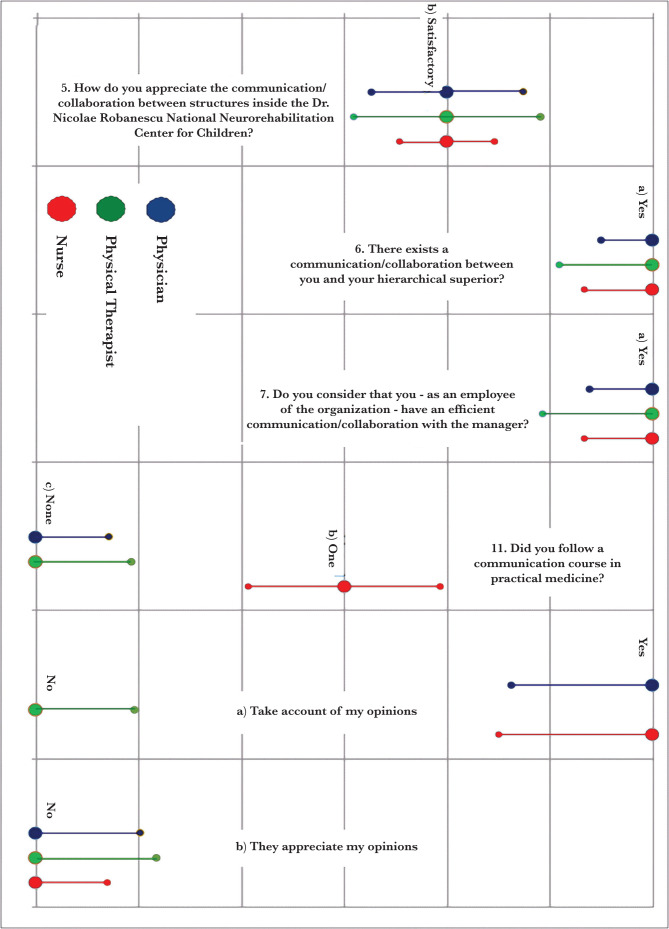
Situation of the groups based on their responses to the study's questions.

For question no. 34, the statistical relevance of each sub-item can be assessed. The weights associated with the sub-items in question no. 13 are as follows: there were no statistical relevances higher than 0.1431, and this value is too low to say that the groups would differ based on the responses to (any of the sub-items from) this question. Figure 4 shows an overview of the situation of the sets based on the answers to these questions – created using medians and ordinal dispersions – highlighted the lack of variability between groups (Tables 12–14).

**Table 12.** Listening and analyzing the proposals of the professional categories by the supervisor and the management team.

Group	**a) They consider my suggestions**	**b) They appreciate my opinions**	**c) They agree with my ideas**	**d) They listen to me**	**e) Sometimes**	**f) No**
**Physician**	9	3	1	4	1	0
**Physical Therapist**	3	4	1	3	3	1
**Nurse**	8	2	4	3	1	0
**Total**	**20**	**9**	**6**	**10**	**5**	**1**

**Table 13.** Evaluation of the statistical relevance regarding the listening and analysis of ideas by the supervisor and the management team.

**a) They consider my suggestions**	**b) They appreciate my opinions**	**c) They agree with my ideas**	**d) He listens to me**	**e) Sometimes**	**f) No**
0.1431	0.0189	0.0746	0.0091	0.0383	0.0450

**Table 14.** Percentage distribution of answers regarding listening and analysis of ideas by the supervisor and the management team.

**a) They consider my suggestions**	**b) They appreciate my opinions**	**c) They agree with my ideas**	**d) He listens to me**	**e) Sometimes**	**f) No**
27.34%	3.61%	14.25%	1.73%	7.32%	8.59%

## Discussion

Interdisciplinary communication is the most effective way to deal with a difficult issue. Communication and consultation within the interdisciplinary team have an impact on both its members and the quality of the medical treatment, which is an essential factor in achieving employee and patient satisfaction. It must be a key ability in every professional’s repertoire [11]. The collaboration of specialists increases patients’ trust in medical services.

Medical organizations aim to deliver high-quality and safe care to the people they serve. Health units must pay special attention to communication between specialists through managerial policies that facilitate regular training in the field of health communication. In order to assure the quality of health services, Romanian health units must first go through the certification procedure and be evaluated by the national health accreditation body of the health units.

By obtaining the accreditation certificate, they demonstrate that they are committed to providing high-quality medical and hotel care services that meet the needs and expectations of the beneficiaries, using all their resources (human, financial, material, and informational) [12]. As a result, medical teams’ communication develops through educational programs, courses, and periodic training according to professional development budgets. Recent studies show that there are no significant differences after communication training between two groups of medical staff, one virtually the other face-to-face (only in terms of costs – these being lower for the virtual version) [13].

By organizing internal communication actions according to specified objectives, effective managerial policies contribute to the enhancement of multidisciplinary communication in a health unit and, implicitly, of the overall medical treatment. Process failures and human disasters are avoided by distributing available specialists, as well as maintaining the motivation of stable medical teams [12], facilitating permanent and transparent communication through a high-performance information system, optimizing information channels, and sharing medical experiences among different specialists.

Communication between different groups of professionals is essential for implementing quality systems at all levels of the organization and for the maximum efficiency of the health unit. The feedback of patients who received medical services demonstrates the level of satisfaction with their needs and the quality of the services provided.

## Conclusion

The role of policies in health communication is to ensure that the population receives high-quality health services and care so that the outcome has the expected impact on the health of the individual and the population. Because the results show that there are no significant differences between the groups, we cannot conclude that the three groups respond statistically differently. We intend to expand the study group in the future in order to increase the statistical significance.

## Acknowledgments

### Ethical approval

The approval for this study was obtained from the Ethics Committee of the Dr. Nicolae Robanescu National Neurorehabilitation Center for Children, Bucharest, Romania (approval ID: 7514/25.09.2019).

### Consent to participate

Written informed consent was obtained from the patient’s parents.

### Conflict of interest

The authors declare that there is no conflict of interest.
